# Mepiquat chloride inhibits soybean growth but improves drought resistance

**DOI:** 10.3389/fpls.2022.982415

**Published:** 2022-09-06

**Authors:** Xiyue Wang, Qi Zhou, Xin Wang, Shuang Song, Jun Liu, Shoukun Dong

**Affiliations:** ^1^College of Agriculture, Northeast Agricultural University, Harbin, China; ^2^Lab of Functional Genomics and Bioinformatics, Institute of Crop Science, Chinese Academy of Agricultural Sciences, Beijing, China

**Keywords:** mepiquat chloride, soybean, drought resistance, flavonoid metabolism, molecular mechanism

## Abstract

Soybeans are an important economic crop. As the most widely used growth regulator globally, the molecular mechanism of mepiquat chloride (DPC) in soybean remains unknown. In this study, RNA sequencing technology combined with ultra-performance liquid chromatography and tandem mass spectrometry were used to analyze the changes in the leaf transcriptome and metabolomics of soybean leaves at the seedling stage under DPC stress. The results showed that differentially expressed genes related to photosynthesis and cell wall synthesis were significantly downregulated at the transcriptional level. In addition, the syntheses of gibberellin, zeatin, brassinolide, and other plant hormones were inhibited in the signal transduction pathway of plant hormones, thereby inhibiting plant growth. In contrast, at the metabolic level, the expression levels of flavonoid differential metabolites were significantly increased, and the proportions of flavonoids in the two varieties were 61.5 and 66%, respectively. The combined analysis of transcriptome and metabolomics showed that the differential expressed genes and metabolites were mainly enriched in the isoflavonoid biosynthesis and flavonoid biosynthesis pathways. Principally, DPC inhibited plant growth but improved drought resistance. Our study is the first to report the molecular mechanism of DPC regulation in soybean, providing useful insights into the rational application of DPC in soybean.

## Introduction

As a food and oil crop of economic significance, soybean (*Glycine max* L. Merr.) is the principal source of plant proteins and oils (Liu et al., 2020). For the most part, soybean taming is thought to have started in East Asia. Although East Asia enjoys verifiable benefits in soybean production, the United States has become the world’s biggest producer since the 1950s (Li et al., 2020). In 2018, the United States created approximately 35% of the world’s reaped soybeans (FAO, 2018). Improvements in yield and quality are currently being pursued, and improving yield and wider adaptive breeding are the main goals of soybean crop improvement (Maranna et al., 2021). However, ensuring the normal growth and development of soybeans is a prerequisite for high yield. Soybean growth and development are usually subjected to different natural tension-imposed abiotic stress (such as dry spells, flooding, saltiness, and weighty metals), which significantly reduces crop yield and quality (Deshmukh et al., 2014; Ishibashi et al., 2018). Drought is one of the most restrictive variables for soybean yield among these abiotic stressors. The yearly all-out crop yield decrease accomplished by dry spells represents more than 50% of all yield misfortunes caused by all pressure factors (Wang et al., 2021). Wei et al. (2018) reported the effects of drought pressure at various soybean developmental phases on its yield. The seed yield of each plant was significantly reduced by 73–82% in plants subjected to drought pressure during blooming and podding, and the maximum loss of 100 seed weight was approximately 42–48% under drought stress during podding. Therefore, ensuring the normal development of plants and improving their stress resistance can ensure smooth yield formation.

Mepiquat chloride (DPC) is an environmentally friendly plant growth regulator used worldwide. In addition to balancing plant nutrition and reproductive growth, DPC is often used to control plant geometry (Zhao and Oosterhuis, 2000). Zhao et al. (2019) found that DPC reduced plant height, resulting in a lower and more compact canopy. Spraying DPC on leaves also reduced plant height, leaf area, asphalt, and canopy, increasing light interception in the canopy, thereby increasing yield. In recent study, DPC was mainly used in cotton production and formed a relatively complete system (Tung et al., 2020). However, DPC has also been applied to other crops. In the production of non-oilseed sunflowers, spraying DPC can effectively reduce plant height and lodging risks (Taşkın et al., 2017). In maize, DPC resists lodging by increasing the actual stem strength and lignin biosynthesis (Kamran et al., 2018). Wang et al. (2016) found that timely and appropriate application of DPC could effectively inhibit the plant height of alfalfa, shorten the internode length of the main stem, thicken the internode of the main stem, and enhance the lodging resistance.

In addition, some studies have reported the stress resistance of DPC. DPC treatment expanded the groupings of solvent protein, chlorophyll, and free proline in cotton; reduced the malondialdehyde (MDA) level, and helped improve resistance to pressure (Xu and Taylor, 1992; Reddy et al., 1996; Tung et al., 2018). Luo et al. (2010) found that irrigating the root with DPC essentially affected the growth and cold resistance of sweet pepper seedlings, manifested as enhanced superoxide dismutase and catalase activities, which can more effectively eliminate hydrogen peroxide in the body, increase the accumulation of abscisic acid by increasing the content of osmotic regulating substances such as soluble sugar and proline in leaves, thereby alleviating the damage caused by low-temperature stress, manifested as a decrease in relative conductivity and MDA content. Endogenous restraint on gibberellin/gibberellic acid (GA) biosynthesis by DPC advances plants by repressing cell prolongation and decreasing the internode length (Rademacher, 2000). Additionally, DPC affects auxin transport and biosynthesis and affects the expression of *PIN* and *LAX* genes by affecting the expression of *YUC* and *AAO*, indicating that DPC affects the formation of lateral roots by promoting the combination of auxin and other hormones (Wu et al., 2019). Presently, the application of DPC in leguminous plants is less reported and research is limited mostly to morphological and physiological characteristics. Moreover, the molecular mechanism by which DPC regulates soybean growth remains largely unknown.

Multi-omics approaches with high-throughput advancements play significant roles in explaining the development, senescence, yield, and reactions to biotic and abiotic stresses in many crops (Yang et al., 2021). Transcriptome investigation plays a significant role in making sense of genome design and capacity; recognizing hereditary organizations of cells, physiology, biochemical, and natural frameworks; and laying out atomic biomarkers of infections, microbes, and ecological difficulties (Jiang et al., 2015). The most prominent platform in plant metabolomics is liquid chromatography–mass spectrometry because it has benefits such as being rapid and highly responsive and having wide sub-atomic weight window and wide inclusion (Zheng et al., 2021). Chen et al. (2021) studied the overall change in sugarcane internode elongation response to DPC using transcriptome technology and observed that the differentially expressed genes (DEGs) were associated with plant chemical sign transduction and biosynthesis of a few metabolites, suggesting that DPC not only inhibits GA biosynthesis but also affects multiple pathways. Zou et al. (2021) utilized physiology and metabolomics to concentrate on the effect of melatonin on soybean and observed that exogenous melatonin application further developed the cell reinforcement framework and photosynthetic limit of soybean, changed carbon and nitrogen digestion, and advanced the development of soybean. Combining multi-omics approaches can elucidate gene functions and networks under physiological and environmental stresses (Singh et al., 2013). Qin et al. (2022) studied the response mechanism of two kinds of *Lycium barbarum* under salt stress through transcriptomics and metabolomics and revealed the intrinsic reason for the high salt tolerance of *L. barbarum*. By integrating metabolomic and transcriptomic studies, Peng et al. (2021) revealed that flavonoids, abscisic acid, and nitric oxide jointly regulate the frost resistance of *Liriope spicata* and identified the involvement of the flavonoid pathway, abscisic acid biosynthesis, and the genes and metabolites of NO oxidative synthesis to construct a comprehensive network of frost resistance of *L. spicata*.

In this study, DPC was sprayed on soybean seedlings to incorporate changes in the DEGs and metabolites using high-throughput, high responsive RNA-sequencing (RNS-seq) and ultra-performance liquid chromatography-tandem mass spectrometry. Our findings will assist with a better comprehension of the reaction component of soybean to DPC stress and provide theoretical support for the rational application of DPC in soybean.

## Materials and methods

### Plant materials and treatment

Two varieties with significant differences in drought resistance at the seedling stage, the sensitive variety Heinong65 (HN65) and the drought-resistant variety Heinong44 (HN44) were selected as experimental materials and provided by the Northeast Agricultural University. The previous physiological tests showed that HN44 had stronger drought resistance than HN65 (Wang et al., 2021). In this experiment, soybean seeds with full-grain, consistent size, and no disease or insects were selected for sowing using the sand pot method. Six seeds were sown per pot and interseeded when the true leaves were fully unfolded. Three robust and consistent seedlings per pot were tested three times. Before sowing for the full expansion of the opposite euphylla, 500 mL of distilled water was poured once a day; when the leaves were completely expanded, Hoagland nutrient solution was poured once a day, 500 mL each time. When the soybean grew to the seedling stage (V3 stage), the DPC solution (100 mg/L) was evenly sprayed on the leaf surface until the leaf was completely wet without water dripping. After 7 days, the samples were collected and analyzed. Water was sprayed as for the control group.

### Determination of physiological indicators

From each sample, 0.5 g of leaf material was cut and placed into a pre-cooling bowl, with a small amount of quartz sand and 10 ml of pre-cooled phosphate buffer (pH = 7.8, 0.05 mmol/L) and ground in an ice bath. Subsequently, 10 mL of the pulp was centrifuged at 10,000 rpm for 20 min at 25°C and stored at 0–4°C. Activity levels of superoxide dismutase (SOD) and peroxidase (POD) were determined by nitroblue tetrazolium assay and guaiacol assay, respectively. These methods were performed in accordance with the experimental method of Wang et al. (2021). Phytohormones contents were detected by MetWare^1^ based on the AB Sciex QTRAP 6500 LC-MS/MS platform.

### Transcriptomics experimental method

Twelve libraries, considering four examples and three reproduces, were constructed for transcriptome sequencing. RNA extraction, RNA quality appraisal, library development, and assessment were performed by Metware Biotechnology Co., Ltd. (Wuhan, China). The library development pack was an Illumina NEBNext^®^UltraTM RNA Library Prep Kit. The library was sequenced on the Illumina HiSeq stage, and 150 base-matching ends were produced to understand the codes. Channel crude information used fastp v 0.19.3 to get spotless peruses. HISAT v2.1.0 was used to fabricate the file and clean the contrast peruses with the reference genome. The differential articulation between the two gatherings was broken down using DESeq2 v1.22.1, and the P esteem was adjusted using the Benjamini and Hochberg technique. The evaluation conditions for the DEGs were |log2Fold change| ≥ 1 and FDR < 0.05. Differentially communicated qualities were explained to the Gene Ontology (GO) and Kyoto Encyclopedia of Genes and Genomes (KEGG) pathway datasets to acquire comments on the data.

### Experimental methods of metabolomics

Test readiness, removal examination, metabolite distinguishing proof, and measurement were performed by Metware Biotechnology Co., Ltd., as per the standard methods. These methodologies have been exhaustively described by Yuan et al. (2018), Zhang S. et al. (2019), and Zhang X. et al. (2019). Orthogonal partial least squares discriminant analysis (OPLS-DA) consolidates the OSC and PLS-DA techniques, breaking down the X lattice data into two types of data connected with Y and immaterial to Y and screening the distinction factors by eliminating the unessential contrasts. Celebrity esteem was extracted from the OPLS-DA results and created using the R bundle MetaboAnalystR. The information is intermediate before the logarithmic change (log_2_) and OPLS-DA. A change test (200 stages) was performed to avoid extreme fitting. The metabolites were essentially controlled between still up in the air by VIP ≥ 1 and outright log2FC (various changes) ≥ 1. Principal component analysis (PCA) was performed using measurable capacity prcomp in R. The KEGG compound information base was used to clarify the distinguished metabolites and guide the clarified metabolites to the KEGG pathway dataset. PCA used R (base bundle) 3.5.0, and OPLS-DA used MetaboAnalyst R (R) 1.0.1.

### Statistical analysis

A one-way ANOVA was used to determine the effects of the various sulfur concentrations on soybean yield within each variety. The R software was used to make such ANOVA.

### Real-time quantitative PCR analysis of genes

RNA was extracted according to the method of 2.3. The total RNA was reversely transcribed into cDNA (PrimeScript TM 1st stand cDNA Synthesis Kit) using the kit. The primers for quantitative PCR (qPCR) were shown in Supplementary Material File 5. In addition, the PCR procedure was as follows: initial denaturation at 95°C for 5 min, then 40 amplification cycles were performed at 95°C for 15 s and 60°C for 30 s. The amplification of each sample was tested for three biological replicates, and *CYP2* was used as the control gene. To confirm the specificity of PCR reaction, melting curve was analyzed. In addition, the relative expression of genes was calculated by 2 ^–ΔΔCT^ method.

## Results

### Physiological changes of soybean after spraying with mepiquat chloride

**FIGURE 1:**
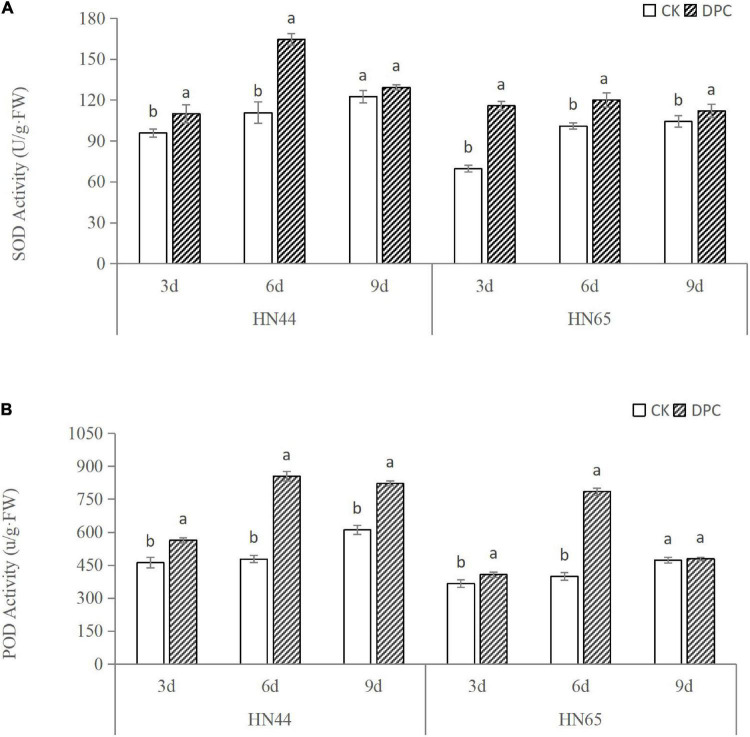
Changes of soybean antioxidant enzyme activities after spraying DPC. Each histogram is the mean of *n* = … Vertical bars are ± 1 S.E. Within each variety, different letters indicate significant differences at *P* < 0.05.

We measured changes in SOD and POD activity in two soybean cultivars within 3–9 days after spraying with DPC (Figure 1). With the increase of DPC spraying days, the SOD and POD activities of the two varieties increased, generally showing a trend of first increasing and then decreasing, and the enzyme activity reached the highest around the sixth day. On the whole, the antioxidative enzyme activities of the two soybean varieties were significantly improved after spraying DPC, and the enzyme activities of HN44 were basically higher than those of HN65 under different days, which confirmed that HN44 had stronger drought resistance.

**FIGURE 2:**
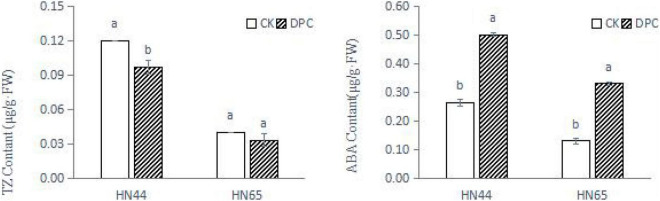
Changes of soybean hormone content after spraying DPC. Each histogram is the mean of *n* = … Vertical bars are ± 1 S.E. Within each variety, different letters indicate significant differences at *P* < 0.05.

We measured hormone content of both varieties on the day of sampling (Figure 2). The content of zeatin decreased in both varieties, significantly decreased in HN44, and slightly decreased in HN65, which was consistent with the inhibition of zeatin synthesis genes found at the transcriptional level. In addition, the ABA content was significantly increased between the two cultivars after spraying with DPC, further confirming the improvement of stress resistance.

### Sequencing quality statistics

Twelve RNA-Seq libraries were constructed and sequenced. The aftereffects of RNA-Seq are summarized in Table 1 and were arranged from 12 libraries. After bad quality information was erased, every library created 5.357−6.974 million clean readings. GC content of every one of the 12 libraries was higher than 44%. Over 92.9% of the clean reads had scores more noteworthy than Q30 in every library. Clean peruses (90.64–92.31%) were planned for the reference genome.

**TABLE 1:** Quality statistics of sequencing samples.

Sample	Raw reads	Clean reads	Q30 (%)	GC content (%)	Reads mapped
HN44CK-1	62,105,260	59,979,664	92.96	45.89	58,143,932 (96.94%)
HN44CK-2	67,513,174	64,867,836	93.47	46.03	62,907,737 (96.98%)
HN44CK-3	65,716,316	63,920,810	93.51	45.49	62,125,671 (97.19%)
HN44DPC-1	68,657,514	66,087,182	93.61	44.83	64,175,752 (97.11%)
HN44DPC-2	55,476,004	53,574,800	94.03	44.8	51,975,025 (97.01%)
HN44DPC-3	64,423,540	61,667,854	93.74	45.36	59,839,451 (97.04%)
HN65CK-1	62,867,210	61,113,810	93.46	45.47	59,147,951 (96.78%)
HN65CK-2	58,240,156	56,365,496	93.56	45.37	54,479,207 (96.65%)
HN65CK-3	60,469,598	58,581,334	93.87	45.26	56,669,150 (96.74%)
HN65DPC-1	69,513,582	63,280,344	94.38	44.64	61,203,657 (96.72%)
HN65DPC-2	76,822,344	69,741,272	94.32	44.25	67,462,020 (96.73%)
HN65DPC-3	71,375,596	65,381,284	94.3	44.87	63,215,354 (96.69%)

### Identification of differentially expressed genes

**FIGURE 3:**
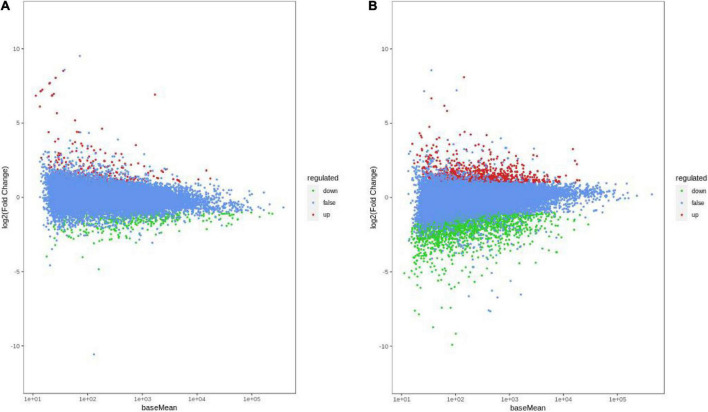
MA Chart of DEGs. The vertical axis represents the log2FC value, which represents the logarithm of the difference fold (log2 Fold_Change); the horizontal axis represents the average value of gene expression in the two samples; the red points represent the up-regulation of gene expression, the green points represent the down-regulation, and the blue Indicates that there were no significant differences in gene expression. **(A)** HN44 group; **(B)** HN65 group.

To better understand the molecular basis of HN44 and HN65 in the DPC reaction, we performed transcriptome sequencing and analyzed DEGs in HN44 and HN65 under control and DPC conditions. As shown in Figure 3, compared with the control group, 717 DEGs were detected in the HN44CK vs. HN44DPC group after DPC spraying, of which 219 DEGs were upregulated and 418 DEGs downregulated. A total of 2,880 DEGs were detected in the HN65CK vs. HN65DPC group, of which 828 DEGs were upregulated and 2,052 DEGs downregulated. In general, compared with HN44, HN65 had more number of DEGs. After spraying with DPC, the downregulated genes of the two varieties were greater than the upregulated genes, and DPC inhibited the transcription levels of the two soybean varieties at the seedling stage.

### Kyoto encyclopedia of genes and genomes analysis

**FIGURE 4:**
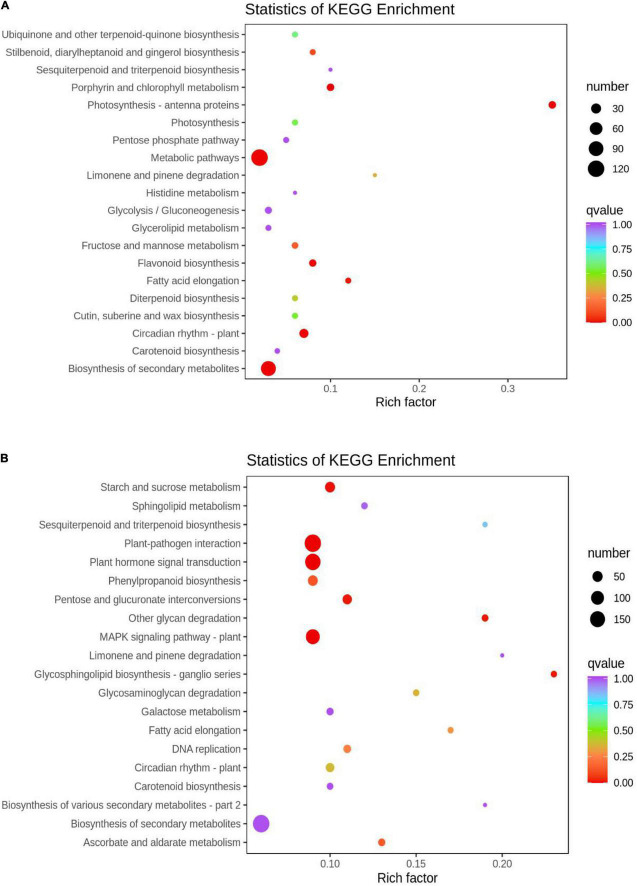
KEGG enrichment scatter plot. The ordinate represents the KEGG pathway. The abscissa represents the Rich factor. The greater the Rich factor, the greater the degree of enrichment. The larger the dot, the higher the number of differential genes enriched by the pathway. The redder the color of the dots, the more significant the enrichment. **(A)** HN44 group; **(B)** HN65 group.

The functions of the DEGs between the two groups were determined by KEGG analysis. The DEGs in the HN44-CK vs. HN44-DPC group (Figure 4A) were mainly enriched in photosynthesis-antenna proteins, biosynthesis of secondary metabolites, circadian rhythm-plant, porphyrin and chlorophyll metabolism, metabolic pathways, and other pathways. In the HN65-CK vs. HN65-DPC group (Figure 4B), the DEGs were mainly involved in plant hormone signal transduction, plant-pathogen interaction, MAPK signaling pathway, starch and sucrose metabolism, and other glycan degradation. The pentose and glucuronate interconversions were also enriched. Notably, in the metabolic pathways of the two varieties, glycolysis and pentose phosphate pathways were inhibited and the related genes were downregulated. Some hormone signaling pathways were also suppressed. In addition, the related metabolic pathways of flavonoids and isoflavones were also involved, proving that DPC had an effect on plant resistance.

### Gene ontology enrichment analysis

**FIGURE 5:**
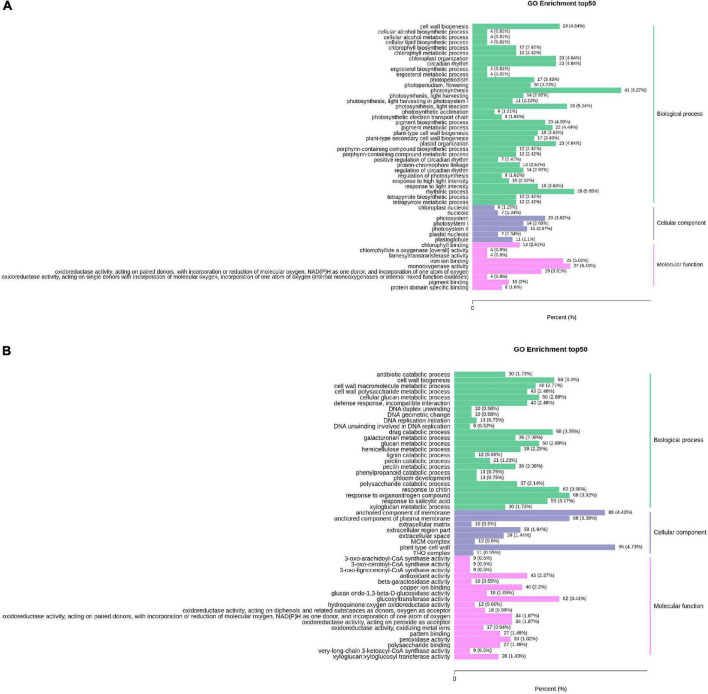
Column chart of GO enrichment of DEGs. The abscissa represents the ratio of genes annotated to the entry to the total number of annotated genes, and the ordinate represents the name of the GO entry. The labels to the right of the graph represent the categories to which the GO entry belongs. **(A)** HN44 group; **(B)** HN65 group.

Figure 5A shows the GO analysis of the DEGs between HN44-CK vs. HN44-DPC. The DEGs were mainly enriched in photosynthesis and cell wall-related pathways in biological processes. The DEGs involved in photosystems, photosystem I, and photosystem II were all downregulated in cellular components. In terms of molecular functions, the DEGs were enriched for chlorophyll-binding, pigment binding, and other processes. Most DEGs were downregulated. Figure 5B shows the GO analysis results for HN65-CK vs. HN65-DPC. In biological processes, the DEGs were mainly enriched in response to chitin, xyloglucan metabolic process, hemicellulose metabolic process, and other pathways and a large number of DEGs were downregulated. In molecular functions, the DEGs were enriched in the xyloglucan:xyloglucosyl transferase activity, glucosyltransferase activity, and copper ion binding. In cell components, the DEGs were mainly enriched in the plant-type cell wall, anchored component of membrane, anchored component of the plasma membrane, and other pathways. In addition, some GA-related pathways were found in both varieties in our study, but most genes were downregulated. In the biosynthesis and metabolism of GA, five genes, *LOC100779318, LOC100790757, LOC100805270, LOC100813185*, and *LOC100793950*, were downregulated in HN44-CK vs. HN44-DPC and seven genes were involved in HN65-CK vs. HN65-DPC. In addition, the genes *LOC100808546, LOC100794180*, and *LOC100801187* were upregulated. The genes *LOC100813185, GMMYB84, LOC100795921*, and *LOC100789097* were downregulated, and GA biosynthesis and metabolism were significantly inhibited. Overall, DPC inhibited plant growth-and development-related pathways, such as photosynthesis and cell wall biosynthesis, thus limiting plant growth.

### Principal component analysis analysis of metabolomic samples

**FIGURE 6:**
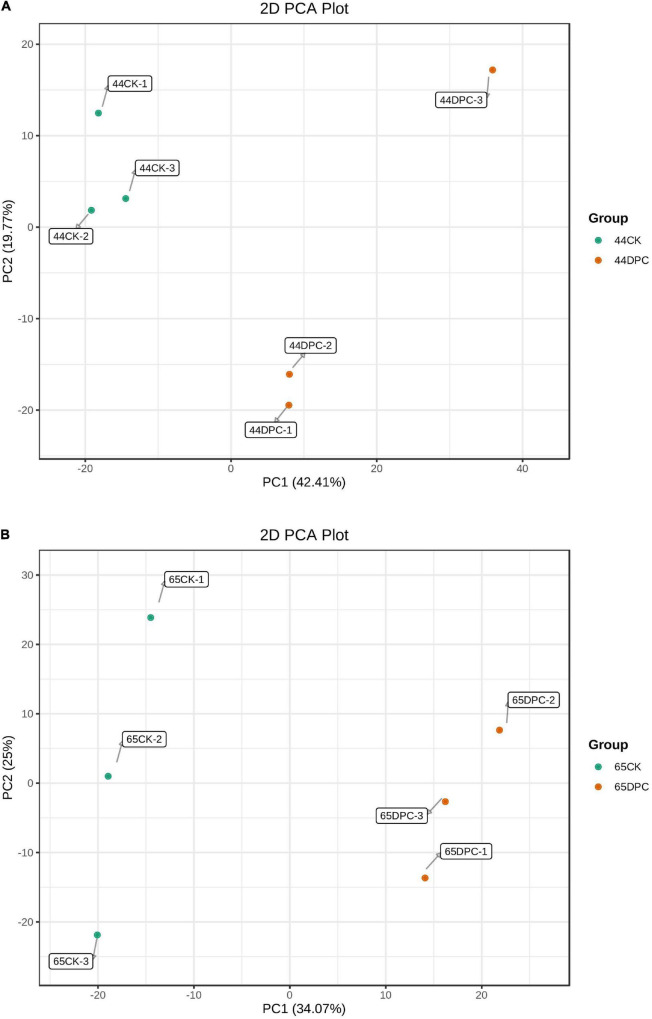
PCA score chart of mass spectrometry data of each sample and quality control sample. PC1 represents the first principal component, PC2 represents the second principal component, and the percentage represents the interpretation rate of this principal component to the dataset; each point in this figure represents a sample, and samples in the same group are represented by the same color. **(A)** HN44 group; **(B)** HN65 group.

PCA was performed on the samples to comprehend the metabolic contrasts among the samples in each group and the two varieties among the samples in the group. The difference between samples in each group was small, whereas the difference between the DPC and CK groups was significant (Figure 6). The first component (PC1) scores for HN44 and HN65 varieties were 42.41 and 34.07%, respectively, and the second component (PC2) scores were 19.77 and 25%, respectively. There was a significant difference between the treatment and control groups, indicating that the plants of the two varieties showed significant metabolic differences after DPC spraying.

### Screening and analysis of differential metabolites (DAMs)

**FIGURE 7:**
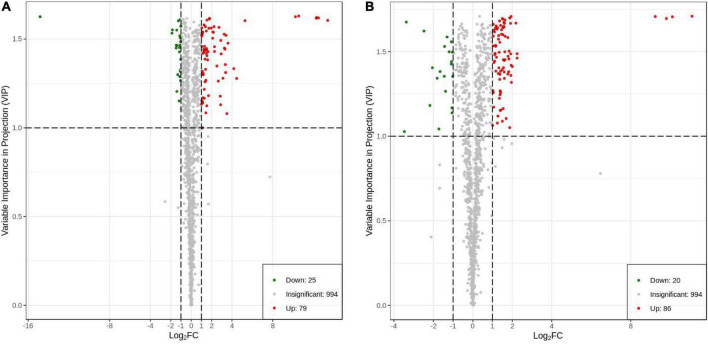
Differential metabolite volcano plot. Each point in the volcano plot represents a metabolite, in which the green point represents the down-regulated differential metabolite, the red point represents the up-regulated differential metabolite, and the gray represents the metabolite detected but not significantly different; the abscissa represents a metabolite In the logarithm of the relative content difference between the two groups of samples (log2FC), the larger the absolute value of the abscissa, the greater the relative content difference of the substance between the two groups of samples. The ordinate represents the VIP value, and the larger the ordinate value, the more significant the difference. **(A)** HN44 group; **(B)** HN65 group.

The fold change was combined with the VIP value of the OPLS-DA model to screen for the DAMs. A total of 104 DAMs (79 upregulated and 25 downregulated; Figure 7A) were generated in HN44-CK and HN44-DPC (Supplementary Table 1), including five amino acids and their derivatives, six phenolic acids, four nucleotides and their derivatives, 64 flavonoids, six lignans and coumarins, eight other classes, three alkaloids, one terpenoid, four organic acids, and three lipids. A total of 106 DAMs (86 upregulated and 20 downregulated; Figure 7B) were generated in HN65-CK and HN65-DPC (Supplementary Table 2), including two amino acids and their derivatives, seven phenolic acids, seven nucleotides and their derivatives, 70 flavonoids, three lignans and coumarins, four other classes, one alkaloid, six terpenoids, one organic acid, and five lipids. In the two varieties, flavonoids accounted for 61.5 and 66% of the total DAMs, respectively, and most substances were upregulated substances, indicating that spraying DPC enhanced the stress resistance of plants to a certain extent.

### Analysis of the top 20 DAMs

**FIGURE 8:**
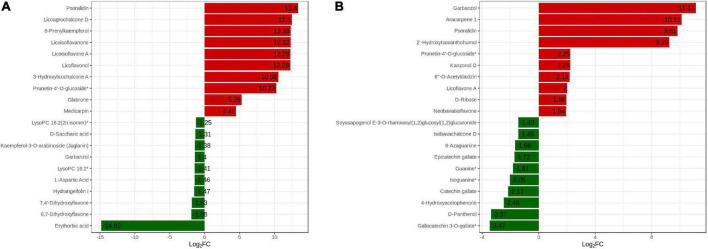
Dynamic distribution of differences in metabolite content in the TOP20. The abscissa is the log2FC of the differential metabolites, and the ordinate is the differential metabolites. Red represents up-regulated differential metabolites and green represents down-regulated differential metabolites. **(A)** HN44 group; **(B)** HN65 group.

We analyzed the changes in the top 20 DAMs between the two cultivars to explain the various effects of DPC on the two cultivars (Figure 8). Among the top 10 substances upregulated, eight flavonoids were included in each of the two varieties. Psoralidin, eurycarpin A, and prunetin-4’-O-glucoside* accumulated in both cultivars. Eurycarpin A and prunetin-4’-O-glucoside* had a large difference in Log2FC values in the two cultivars, and in HN44, the values were 12.29 and 10.23 and only 2.00 and 2.25 in HN65. The psoralidin Log2FC value was the largest in HN44, and the garbanzol Log2FC value was the highest in HN65. Among the top 10-downregulated substances, erythorbic acid was the most downregulated in HN44, and gallocatechin 3-O-gallate* was the most downregulated in HN65. Overall, DPC substantially stimulated flavonoid upregulation.

### Kyoto encyclopedia of genes and genomes enrichment analysis of DAMs

**FIGURE 9:**
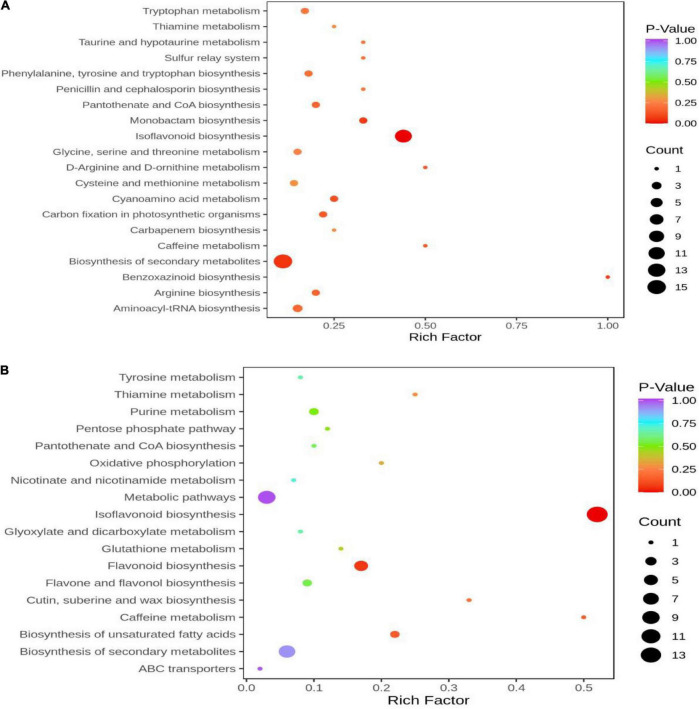
KEGG enrichment map of DAMs. The abscissa represents the Rich Factor corresponding to each pathway, and the ordinate represents the name of the pathway. The color of the point reflects the *p*-value size, and the redder the more significant the enrichment. The size of the dots represents the number of enriched differential metabolites. **(A)** HN44 group; **(B)** HN65 group.

KEGG pathway enrichment analysis was performed to clarify the function of DAMs In HN44-CK vs. HN44-DPC (Figure 9A), The DAMs were enriched in the biosynthesis of isoflavonoid, secondary metabolites, and monobactam and other pathways according to the *P*-value. In HN65-CK vs. HN65-DPC (Figure 9B), the DAMs were enriched in isoflavonoid biosynthesis, flavonoid biosynthesis, caffeine metabolism, and other pathways. Among these, isoflavonoid biosynthesis, flavonoid biosynthesis, and other pathways play a significant role in plant pressure opposition.

### Combined analysis of transcriptome and metabolome

**FIGURE 10:**
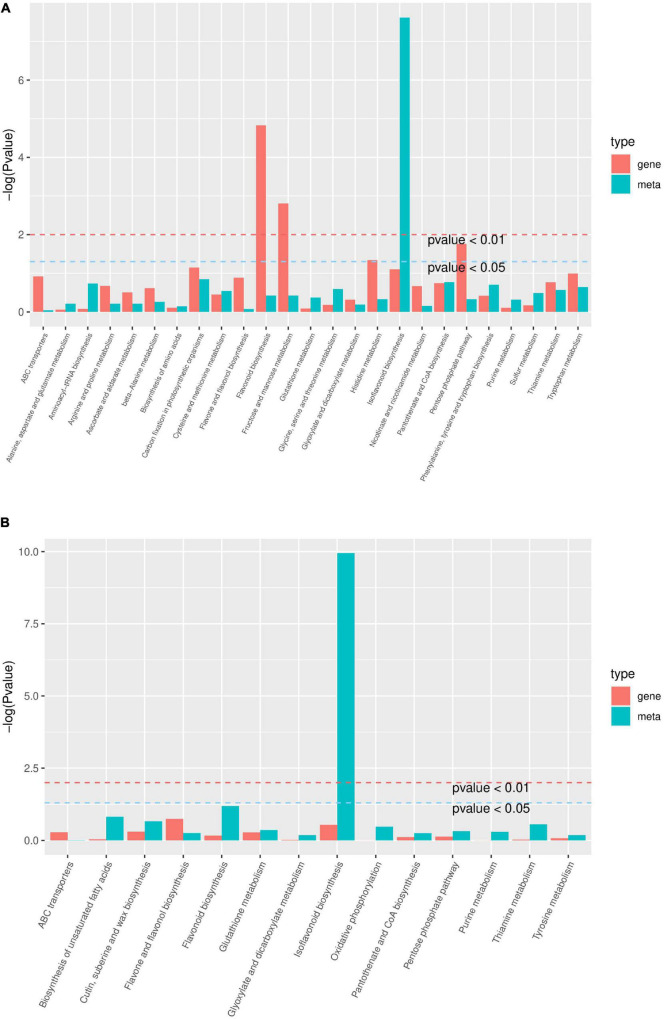
KEGG enrichment analysis *p*-value histogram. The abscissa represents the metabolic pathway, the red in the ordinate represents the enrichment *p*-value of the differential metabolites, and the green represents the enrichment *p*-value of the differential gene, which is represented by -log (*P*-value), the higher the ordinate, the higher the degree of enrichment the stronger. **(A)** HN44 group; **(B)** HN65 group.

In HN44-CK vs. HN44-DPC (Figure 10A), DEGs and DAMs were involved in 28 metabolic pathways, mainly in isoflavonoid biosynthesis, carbon fixation in photosynthetic organisms, pantothenate and CoA biosynthesis, flavonoid biosynthesis, and enrichment of other pathways. In HN65-CK vs. HN65-DPC (Figure 10B), DEGs and DAMs were involved in 14 metabolic pathways, mainly isoflavonoid biosynthesis, flavonoid biosynthesis, biosynthesis of unsaturated fatty acids, and other pathways. Based on the above results, we further analyzed the changes in genes and metabolites in the isoflavonoid and flavonoid biosynthesis pathways in the two cultivars.

### Changes in the DEGs and DAMs in the isoflavone synthesis pathway

**FIGURE 11:**
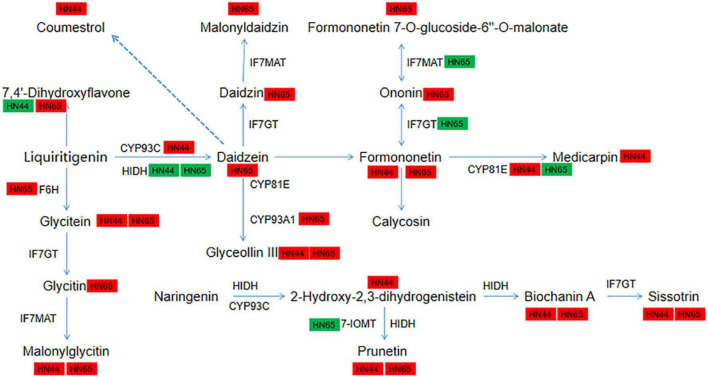
Pathway diagram of isoflavone synthesis. In this figure, red indicates up-regulation of genes/metabolites, and green indicates down-regulation of genes/metabolites.

In HN44-CK vs. HN44-DPC (Figure 11A), most DEGs and DAMs were downregulated, except for *LOC100800199*, which encodes 2-hydroxyisoflavanone dehydratase, and the metabolite 7,4’-dihydroxyflavone appeared to be upregulated. Among them, the log2FoldChange reached 2.37 and 2.27 for gene-*IFS2* and *novel-550* encoding 2-hydroxyisoflavanone synthase, respectively. The upregulation of these genes resulted in the upregulation of their downstream metabolites coumarin, formononetin, calycosin, glycitein, malonylglycitin, prunetin, 2-hydroxy-2,3-dihydrogenistein, biochanin A, and biochanin A-beta-D-glucoside. In addition, the genes *CYP81E11* and *LOC100794610* encoding isoflavone/4’-methoxyisoflavone 2’-hydroxylase upregulated log2FoldChange by 3.22 and 4.62, respectively, and its downstream metabolites medicarpin and glyceollin III were upregulated. In contrast, most genes in HN65-CK vs. HN65-DPC (Figure 11B) were downregulated at the transcriptional level. Except for the gene *CYP71D10*, which encodes flavonoid 6-hydroxylase, and the gene *LOC100805931*, which encodes 3,9-dihydroxypterocarpan 6a-monooxygenase, were upregulated, while the rest of the genes were downregulated. These genes encode 2-hydroxyisoflavanone dehydratase, isoflavone-7-O-methyltransferase, isoflavone 7-O-glucosyltransferase, isoflavone 7-O-glucoside-6″-O-malonyltransferase, isoflavone/4′-methoxyisoflavone 2′-hydroxylase, isoflavone 7-O-glucosyltransferase, and isoflavone 7-O-glucoside-6”-O-malonyltransferase. However, HN65 had more upregulated metabolites at the metabolic level than HN44, including 7,4’-dihydroxyflavone, formononetin, daidzein, formononetin 7-O-glucoside, formononetin 7-O-glucoside-6’-O-malonate, malonyldaidzin, and malonylglycitin. Although the two varieties were downregulated to varying degrees at the transcript level, most of the metabolites were upregulated at the metabolic level. 7,4’-Dihydroxyflavone was downregulated in HN44 and upregulated in HN65. Malonylglycitin, formononetin, glyceollin III, prunetin, and biocharanin A were the core metabolites of soybean that responded to DPC.

### Changes in the DEGs and DAMs in the flavonoid synthesis pathway

**FIGURE 12:**
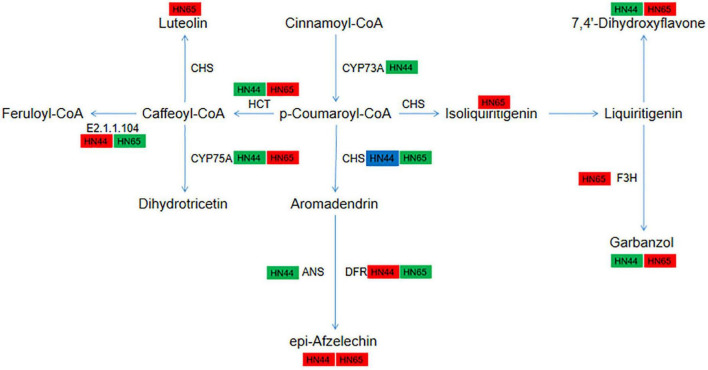
Pathway diagram of flavonoid synthesis.

The flavonoid biosynthesis pathways of the two varieties mainly differed at the gene level (Figure 12). In HN44-CK vs. HN44-DPC, the gene *LOC100806561* encoding shikimate O-hydroxycinnamoyltransferase and the genes *LOC100783085* and *LOC100787507* encoding flavonoid 3’,5’-hydroxylase were downregulated. The gene *LOC100820497* encoding anthocyanidin synthase was downregulated. On the contrary, the gene *LOC100787267* encoding flavanone 4-reductase, genes *LOC100500657* and *LOC100776619* encoding caffeoyl-CoA O-methyltransferase, and gene *LOC100787267* encoding bifunctional dihydroflavonol 4-reductase were upregulated. In addition, the metabolites 7,4’-dihydroxyflavone and arbanzol, were downregulated and the metabolite epi-afzelechin was upregulated. In HN65-CK vs. HN65-DPC (Figure 12B), unlike HN44, the genes *LOC100779814* and *LOC100814459* encoding shikimate O-hydroxycinnamoyltransferase were upregulated, and the gene *LOC100783085* encoding flavonoid 3’,5’-hydroxylase was upregulated. The gene *LOC100804211*, which encodes bifunctional dihydroflavonol 4-reductase, was downregulated. The expression of genes in these pathways showed opposite trends between the two varieties. The gene *LOC100780057* encoding naringenin 3-dioxygenase was upregulated, and five metabolites were identified: luteolin, epi-afzelechin, garbanzol, 7,4’-dihydroxyflavone, and isoliquiritigenin.

### Association analysis of the DAMs and DEGs

**FIGURE 13:**
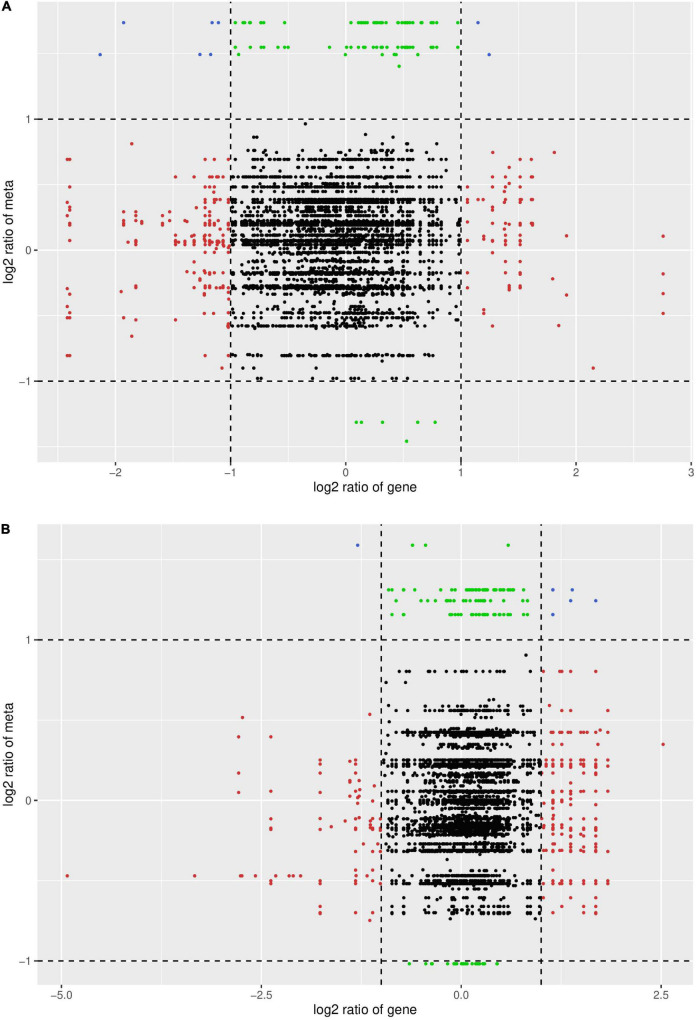
Nine-quadrant plot of correlation between DAMs and DEGs. The abscissa represents the log2FC of genes, and the ordinate represents the log2FC of metabolites. The 1st, 2nd, and 4th quadrants indicate that metabolites are expressed at higher levels than genes; the 3rd and 7th quadrants indicate that the differential expression patterns of genes and metabolites are consistent; the 5th quadrant indicates that neither genes nor metabolites are differentially expressed; The 9th quadrant indicates that metabolites are expressed in lower abundance than genes. **(A)** HN44 group; **(B)** HN65 group.

Correlation analysis was performed on the DAMs and DEGs, and those with Pearson correlation coefficient results greater than 0.8 were selected. Nine quadrant maps and correlation coefficient cluster heat maps were drawn. As shown in Figure 13, in the two varieties, the most abundant DAMs and DEGs were in the second, fourth, and sixth quadrants. They were negatively correlated in the second, fourth, and sixth quadrants, while the upper metabolites were upregulated and the genes were unchanged or downregulated. In the sixth quadrant, the genes were upregulated and the metabolites were unchanged or downregulated. Overall, the transcription level was suppressed after spraying with DPC and the metabolic level increased.

### Quantitative real-time PCR

**FIGURE 14:**
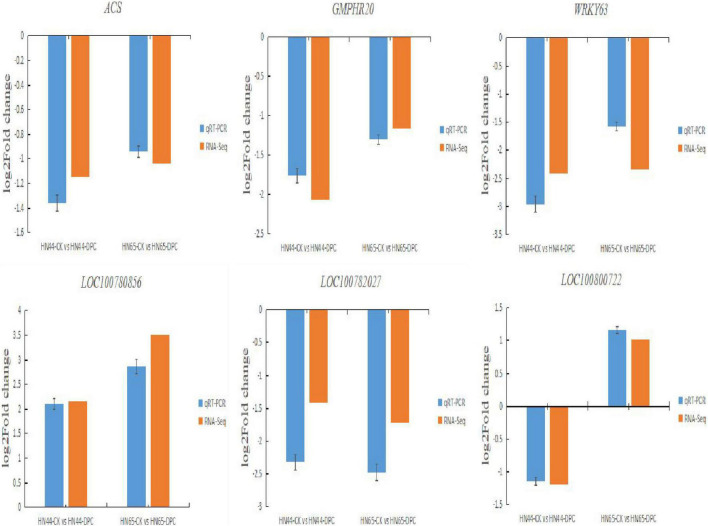
Verification of RNA-seq results by qRT-PCR.

qRT-PCR was used to validate RNA-seq results (Figure 14). We selected 6 important genes for analysis. These genes are mainly involved in amino acid metabolism, plant hormone transmission and so on. Six tested genes *ACS, GMPHR20, WRKY63, LOC100800722, LOC100782027, LOC100780856* showed similar qPCR results to RNA-seq. These results show the high reliability of RNA-Seq data.

## Discussion

DPC is one of the most widely used synthetic substances for managing plant development. However, its molecular mechanism in soybeans has not yet been reported. In previous reports, DPC inhibited cotton cell elongation by downregulating GhEXP and GhXTH2 and reduced endogenous GA accumulation by inhibiting GA biosynthesis and metabolic genes (Wang et al., 2014). DPC limits plant growth in soybean by inhibiting the plant growth and development-related pathways, such as photosynthesis and cell wall biosynthesis. Wang et al. (2020) found that DPC decreases internode prolongation by repressing cell division and development; DPC significantly reduced the number and length of internode cells, and numerous genes related to the cell cycle and cell divider were changed by DPC treatment. This finding was similar to that of the present study. The DEGs involved in cell wall-related items were significantly downregulated in both varieties. In a study by Chen et al. (2021) on sugarcane, the gene changes between the control and DPC treatment were primarily 6 days after splashing. After 10 days, the effect of DPC on gene expression was reduced. They observed that the gene expression of DPC did not have an intense impact, and it appeared that DPC caused a change in gene expression after 4–6 days. Wang et al. (2014) found that the contents of endogenous bioactive GAs (GA3 and GA4) recorded after DPC treatment for 6 days significantly decreased by 30.4 and 43%, respectively Therefore, the samples collected on the seventh day were selected for analysis to ensure our experiment’s reliability.

DPC application also affects plant photosynthesis and has also been reported to alter the distribution of photosynthates, CO_2_ absorption, and fixation (Tung et al., 2018). However, this effect was not a single promotion or inhibition. Gonias et al. (2012) showed that DPC could improve the single-leaf Pn and light use efficiency of cotton. However, recent reports showed that with an increase in DPC dosage, photosynthesis decreased by 1–28%, and lint yield decreased by 6–29% (Tung et al., 2018). This may be due to the difference in the DPC dosage. A previous study showed that low concentrations of DPC could promote cotton growth, regulate cotton vegetative growth, and improve cotton resistance. However, high doses of DPC can significantly inhibit cotton growth (Xiaoxia et al., 2013). In soybeans, the application of 100 mg/L DPC inhibited photosynthesis. According to RNA-Seq data, genes related to photosynthesis (including those related to chlorophyll binding, pigment binding, photosynthetic system I, and photosynthetic system II) were downregulated in the two cultivars. Overall, the photosynthetic process is inhibited, but physiological tests are still needed to verify it, which is one of the shortcomings of this experiment.

Plant growth hormones, such as auxins, cytokinins, brassinolides, GA, and strigolactones, play important roles in plant development (Durbak et al., 2012). In addition to GA, we found that DPC altered the biosynthesis and signal transduction of multiple hormones. This change seems to have a greater impact on the sensitive varieties. For example, in HN65, many genes involved in tryptophan metabolism were downregulated and all genes in the auxin-responsive GH3 gene family were downregulated. Their function is to regulate cell and plant growth. First, some genes were downregulated during zeatin biosynthesis, inhibiting cell division. Second, the biosynthesis-related genes of diterpenoids and brassinosteroids were inhibited. These hormones mainly regulate cell elongation, cell division, and stem growth, and the downregulation of gene expression significantly inhibits plant growth. In physiological indicators, we also detected the changes of TZ and ABA content. After spraying with DPC, ABA accumulated between the two cultivars, and an increase in ABA content was also detected at the metabolic level. Generally, plants can adapt to drought stress by controlling ABA content by upregulating the expression of ABA biosynthesis and ABA signaling genes (Zhang Z. et al., 2019). The increase in ABA content also represented a certain degree of increase in stress resistance.

A few studies have reported that DPC can enhance stress resistance. DPC has good systemic conductivity and can be freed in cell biofilms, thereby scavenging free radicals in the cell. Simultaneously, it also affects the antioxidant system, resulting in enhanced activity of antioxidant enzymes (Zhao et al., 2017). However, the physiological response of soybean to DPC is rarely reported. We examined the changes in the activities of the two antioxidant enzymes after spraying DPC, and the activities of SOD and POD were significantly increased after 6 days of DPC spraying. This result also represents that DPC promotes the improvement of antioxidant levels. Liu et al. (2015) indicated that spraying DPC at low temperatures could increase the free proline content in the leaves of *Phoebe bournei* seedlings, which was conducive to improving the defense ability of plants against low-temperature stress. In this experiment, according to the joint analysis of transcriptome and metabolomics results, isoflavonoid and flavonoid biosynthesis were the main enrichment pathways of the DEGs and DAMs. In particular, at the metabolic level, many flavonoid metabolites were upregulated in the two varieties and were more abundant in HN65. We found that DPC could regulate several key pathways, such as isoflavonoid, flavonoid, flavone, and flavonol biosyntheses, which were enriched. In plants, flavonoids generally exist in seeds, blossoms, and leaves. As secondary metabolites, plant flavonoids play a significant role in controlling plant responses to natural and abiotic ecological variables (Shen et al., 2022). Accumulation of flavonoids is beneficial for plants to resist abiotic stress (Petrussa et al., 2013). In our previous study, metabolomic changes in these two varieties after drought stress at the flowering stage were analyzed (Wang et al., 2022). Four flavonoids accumulated in HN44 and seven in HN65. Drought-related substances, such as luteolin and catechin, were also detected. Ahmed et al. (2021) also reported an increase in phenols, flavonoids, anthocyanins, and carotenoids under drought stress. Therefore, spraying DPC on the leaf surface at the seedling stage could improve drought resistance, particularly in sensitive varieties. In this experiment, we investigated the physiological and molecular mechanisms of soybean response to DPC, which inhibits soybean growth by inhibiting photosynthesis, hormone synthesis, cell wall synthesis, etc. In addition, DPC could potentially improve drought resistance, manifested as an increase in antioxidant enzyme activity. DPC significantly affected the synthesis and metabolism of flavonoids. However, in order to fully construct the network of DPC regulating soybean growth and development, experiments, including proteomics and more morphological and physiological indicators, are still needed.

## Conclusion

In summary, we conducted transcriptome and metabolomic analyses to better understand the molecular mechanisms of DPC in soybean. We found that DPC inhibits plant growth by inhibiting photosynthesis and cell wall synthesis. In addition, DPC changed the biosynthesis and signal transduction of GA and the biosynthesis and signal transduction of other plant hormones, including zeatin and brassinolide. DPC also promoted the accumulation of flavonoids, resulting in increased drought resistance. In the future, we will further analyze the mechanism by which DPC regulates soybean drought stress.

## Data availability statement

The datasets presented in this study can be found in online repositories. The names of the repository/repositories and accession number(s) can be found below: https://www.ncbi.nlm.nih.gov/, PRJNA823397 and PRJNA854192.

## Author contributions

XYW: original manuscript writing. QZ and SS: software analysis. XW: investigation. JL and SD: manuscript editing and review. All authors contributed to the article and approved the submitted version.

## Footnotes

1
http://www.metware.cn/

## Abbreviations

DPC: mepiquat chloride
GA: gibberellin/gibberellic acid
GO: Gene Ontology
Hn44: Heinong44
HN65: Heinong65
KEGG: Kyoto Encyclopedia of Genes and Genomes
MDA: malondialdehyde
OPLS-DA: Orthogonal partial least squares discriminant analysis
PCA: Principal component analysis
RNS-seq: RNA-sequencing.
