# Corrigendum: Mepiquat chloride inhibits soybean growth but improves drought resistance

**DOI:** 10.3389/fpls.2022.1068683

**Published:** 2022-11-25

**Authors:** Xiyue Wang, Qi Zhou, Xin Wang, Shuang Song, Jun Liu, Shoukun Dong

**Affiliations:** ^1^College of Agriculture, Northeast Agricultural University, Harbin, China; ^2^Lab of Functional Genomics and Bioinformatics, Institute of Crop Science, Chinese Academy of Agricultural Sciences, Beijing, China

**Keywords:** mepiquat chloride, soybean, drought resistance, flavonoid metabolism, molecular mechanism

In the published article, there were two errors in **Figure 11** and **Figure 12**. The contents of the two Figures were reversed, and the caption for **Figure 12** mistakenly included "**(A)** HN44 group; **(B)** HN65 group.". The corrected **Figure 11** and **Figure 12** their captions appear below.

**Figure 11:**
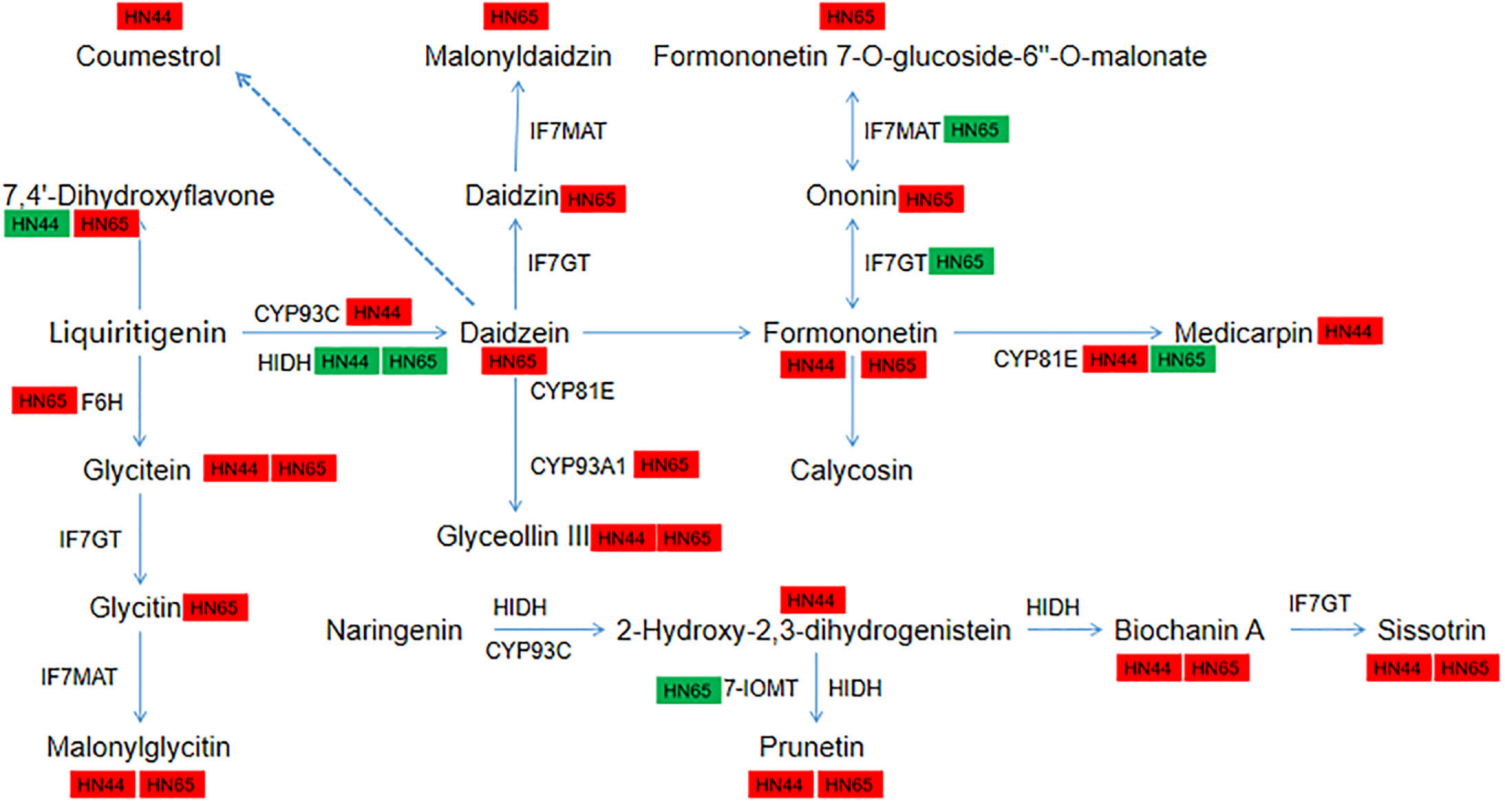
Pathway diagram of isoflavone synthesis. In this figure, red indicates up- regulation of genes/metabolites, and green indicates down-regulation of genes/metabolites.

**Figure 12:**
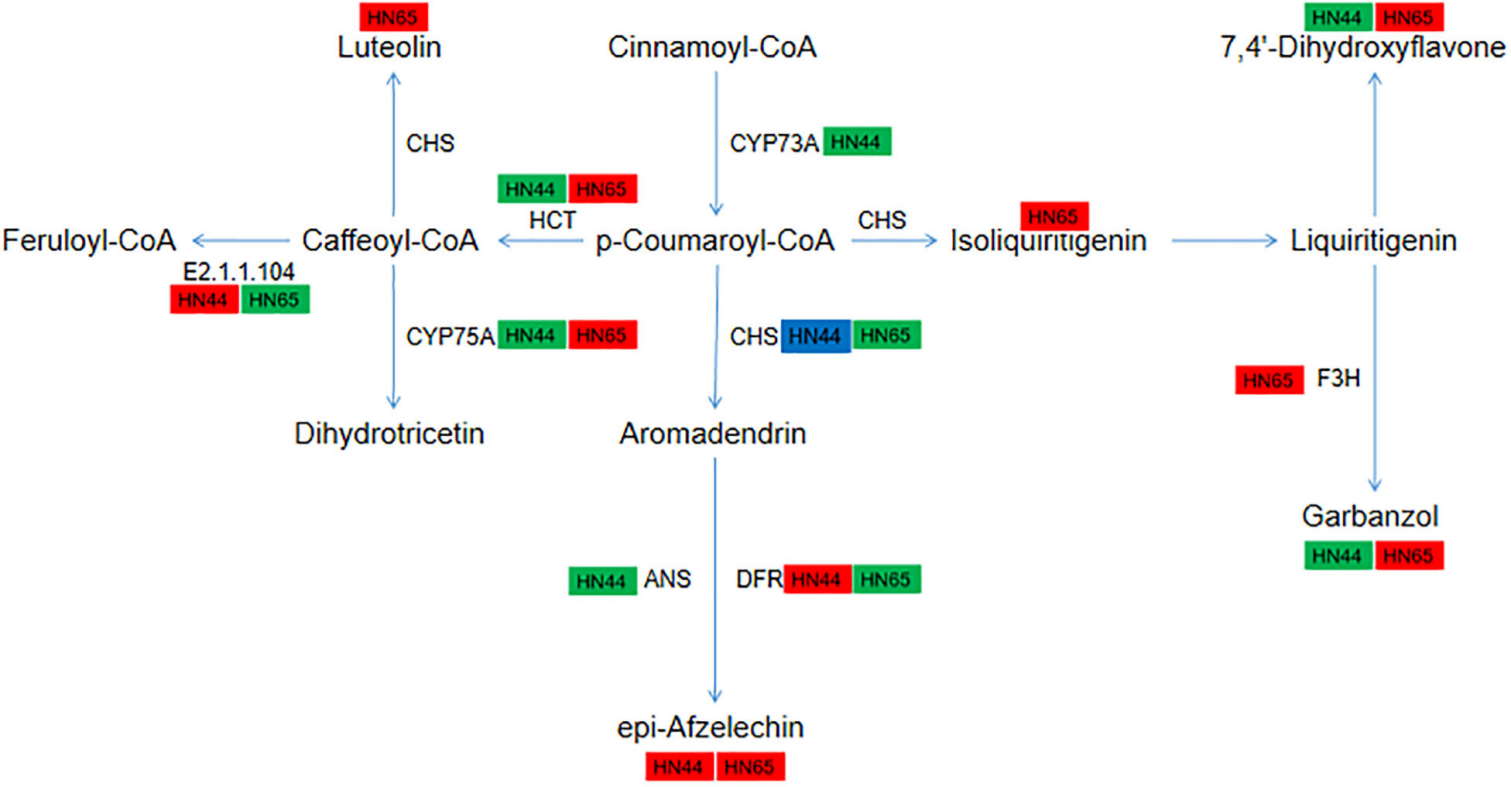
Pathway diagram of flavonoid synthesis.

In the published article, the name of a gene was misspelled in the **Results**, *Changes in the DEGs and DAMs in the isoflavone synthesis pathway*. This sentence previously stated: “Among them, the log2FoldChange reached 2.37 and 2.27 for gene-IFS2 and novel 0.550 encoding 2-hydroxyisoflavanone synthase, respectively”. The corrected sentence appears below:

“Among them, the log2FoldChange reached 2.37 and 2.27 for gene-*IFS2* and *novel-550* encoding 2-hydroxyisoflavanone synthase, respectively”.

The authors apologize for these errors and state that this does not change the scientific conclusions of the article in any way. The original article has been updated.

## Publisher’s note

All claims expressed in this article are solely those of the authors and do not necessarily represent those of their affiliated organizations, or those of the publisher, the editors and the reviewers. Any product that may be evaluated in this article, or claim that may be made by its manufacturer, is not guaranteed or endorsed by the publisher.

